# Risk Factors Associated With Unsuccessful Linkage to Outpatient Hepatitis C Care

**DOI:** 10.7759/cureus.58313

**Published:** 2024-04-15

**Authors:** Carlo Foppiano Palacios, Brianna Dubose, Sarah Schmalzle

**Affiliations:** 1 Department of Medicine, Cooper University Hospital, Camden, USA; 2 Departments of Internal Medicine and Pediatrics, University of Maryland Medical Center, Baltimore, USA; 3 Department of Medicine, University of Maryland School of Medicine, Baltimore, USA; 4 Institute of Human Virology, University of Maryland School of Medicine, Baltimore, USA

**Keywords:** social determinants of health (sdhs), hepatitis c virus (hcv) infection, transitions of care, barriers to care, linkage to care

## Abstract

Background

Modern direct-acting antivirals (DAAs) can treat and cure hepatitis C virus (HCV) infection. Treatment of HCV at a population level has the potential to decrease the prevalence of chronic HCV infection and sequela. Unfortunately, many patients fall off the HCV treatment cascade and do not complete HCV treatment. As social determinants of health (SDHs) affect HCV acquisition, we sought to evaluate factors that may limit successful linkage to outpatient HCV care.

Methods

We conducted a case-control study by matching patients who missed and those who attended their outpatient HCV visits in 2018. We matched cases in a 1:1 ratio using propensity scores.

Results

Of 1,539 patients, 161 (10.5%) did not attend their HCV clinic appointment. Factors associated with a missed HCV visit on bivariate testing included identifying as Black (p=0.03), housing instability (p<0.001), transportation difficulty (p<0.001), history of medication non-adherence (p<0.001), and undergoing screening during an inpatient admission (p<0.001). Multivariate testing found transportation difficulty (p<0.001) and inpatient admission (p=0.002) to be associated with missing their HCV appointment. Patients who attended their HCV visit were more likely to be alive by the end of 2018 (p=0.07).

Conclusion

Patients who missed an initial scheduled infectious disease (ID) clinic appointment for HCV treatment had higher rates of housing instability, transportation difficulties, and medication non-adherence. Patients diagnosed with HCV infection should be provided additional support as appropriate to address the social determinants of health that may limit linkage to outpatient HCV care.

## Introduction

An estimated 3.5 million people have chronic hepatitis C virus (HCV) infection in the USA [1]. HCV treatment includes effective, well-tolerated direct-acting antiviral (DAA) therapy that can lead to a virological cure [2]. By treating HCV, the prevalence of infection will decrease, subsequently leading to decreased viral transmission and an overall reduction in new HCV cases [3,4]. The HCV treatment cascade includes several significant steps to achieve sustained virological response (SVR): screening for chronic infection, referral and access to outpatient care, confirmation of HCV diagnosis, liver fibrosis staging, DAA prescription, and achieved SVR [1].

Out of all patients with HCV, half of patients are aware of their diagnosis, but only 40%-50% of those patients are engaged in outpatient HCV care [1,5]. Only 9%-19% of HCV patients complete HCV DAA and achieve SVR [1]. After HCV screening, the subsequent step is establishing HCV, but risk factors for missing an initial HCV evaluation appointment have not been established. Known risk factors associated with the unsuccessful treatment of HCV include speaking a language other than English, alcohol use, substance use history, and unstable housing [6]. As social determinants of health (SDHs) are known to affect HCV acquisition and treatment, we sought to evaluate factors that may affect the successful linkage to HCV outpatient care to guide future interventions and policies to improve transitions of care.

## Materials and methods

Patient population

We conducted a case-control retrospective chart review on patients with a positive HCV screening test (antibody or viral load polymerase chain reaction (PCR)). We included patients who attended or were scheduled for but did not attend their HCV infectious disease (ID) clinic appointment at the Center for Infectious Diseases at the University of Maryland Medical Center in 2018.

Data collection

We collected data on patient demographics, health insurance, and risk factors that may impair outpatient HCV linkage. The risk factors evaluated included substance use, mental health history, transportation difficulties, housing insecurity, history of medication non-adherence, and alcohol abuse based on previous risk factors associated with HCV treatment [6-8]. Alcohol level risk quantification was not possible, given the limited information on the medical record. Patients were marked to have a history of alcohol use if they were reported to have a heavy alcohol drinking history or if they consumed more than one drink for women and two drinks for men per day. Patients were classified as either linked or unlinked into care based on whether they attended their ID clinic appointment during 2018.

Statistical methods

Linked patients were matched to unlinked patients on a 1:1 ratio based on their age at the scheduled appointment date, appointment date, and reported sex. We used propensity scores <1% (including standardized mean differences < 0.1 and variance ratios < 2) to minimize confounders between these groups. Descriptive statistics were used for summary statistics. Fisher's exact tests were performed for bivariate analyses. Multivariate logistic regression analysis was performed by incorporating only factors with a p-value of <0.10 on bivariate analysis. A predictive model was used for the multivariate logistic regression analysis: the factors with the highest p-values were removed until only factors with a p-value of <0.05 remained. The remaining factors were assessed for interaction. Adjusted odds ratios (aOR) were calculated to determine the strength of the association of multivariate analysis. All data analysis was conducted using R version 4.0.2 and Microsoft Excel (Microsoft Corp., Redmond, WA).

The University of Maryland, Baltimore, Institutional Review Board approved this study.

## Results

Demographics

Of 1,539 patients, 161 (10.5%) did not attend their HCV clinic appointment in 2018. These patients were matched in a 1:1 ratio to 161 patients who did attend their scheduled HCV clinic appointment. Ages were similar across both groups, with a mean age of 48.5 years in the unlinked group and 49.8 years in the linked group (p=0.40). There were 55.3% of males (n=89/161) in the unlinked group and 52.8% (n=85/161) in the linked group (p=0.74). Regarding race, there were more Black patients (72.7% versus 60.9%, p=0.03) and fewer White patients (26.7% versus 37.3%, p=0.056) in the linked group compared to the unlinked group. Patients in both groups had similar rates of having public health insurance (p=0.21).

Risk factors

Both groups reported similarly high rates of alcohol use disorder (52.2% versus 60.2%, p=0.18), substance use disorders (85.1% versus 85.1%, p=1.00), and psychiatric diagnosis (71.4% versus 70.8%, p=1.000). On bivariate testing, patients in the unlinked group had higher rates of housing instability (60.9% versus 39.8%, p<0.001), transportation difficulty (65.8% versus 29.8%, p<0.001), and history of medication non-adherence (60.9% versus 36.6%, p<0.001) compared to patients in the linked group (Table 1). On multivariate analysis, transportation difficulty (p<0.001, aOR 95% confidence interval (CI): 0.17-0.51), outpatient testing (p=0.04, aOR 95% CI: 1.01-18.26), and inpatient testing (p=0.002, aOR 95% CI: 0.04-0.46) were associated with linkage to care.

**Table 1:** Characteristics of patients who were not linked to care (group A) and linked to care (group B)

	Group A (unlinked) (N=161)	Group B (linked) (N=161)	p-value
	Number (%)	Number (%)	
Sex			0.74
Female	72 (44.7)	76 (47.2)	
Male	89 (55.3)	85 (52.8)	
Race			
Asian	1 (0.6)	1 (0.6)	1.00
Black	98 (60.9)	117 (72.7)	0.03
Other	2 (1.2)	0 (0.0)	0.48
White	60 (37.3)	43 (26.7)	0.056
Latino ethnicity	0 (0.0)	2 (1.2)	0.48
Public insurance	152 (94.4)	145 (90.1)	0.21
Risk factors			
Alcohol use disorder	84 (52.2)	97 (60.2)	0.18
Substance use disorder	137 (85.1)	137 (85.1)	1.00
Mental health conditions	115 (71.4)	114 (70.8)	1.00
Housing instability	98 (60.9)	64 (39.8)	<0.001
Transportation difficulty	106 (65.8)	48 (29.8)	<0.001
History of non-adherence	98 (60.9)	59 (36.6)	<0.001
Testing site			
Emergency department	3 (1.9)	2 (1.2)	1.00
Inpatient	151 (93.8)	63 (39.1)	<0.001
Outpatient	6 (3.7)	82 (50.9)	<0.001
Outcome			
Treated	0 (0.0)	49 (30.4)	<0.001
Alive through 2018	154 (95.7)	160 (99.4)	0.073

Clinical outcomes

Fewer patients in the unlinked group received HCV DAA in our medical system than the linked group (0% versus 30.4%, p<0.001). By the end of 2018, more patients in the linked group were alive than in the unlinked group (99.4% versus 95.7%, p=0.07).

## Discussion

Patients who missed their scheduled HCV appointments faced significant difficulties. Risk factors contributing to unsuccessful linkage were more common among patients who missed their ID clinic appointments, particularly housing instability, transportation difficulties, and a history of medication non-adherence. Our study highlights the role of social determinants of health (SDHs) in care transitions, particularly within the transition to outpatient HCV care [9]. Patients referred to outpatient HCV care should be screened for barriers to care and provided additional support to help address the factors that may limit their HCV care and improve the likelihood of successful continuity.

Patients who missed their clinic appointments had higher rates of housing instability, transportation difficulties, and medication non-adherence on bivariate testing, but only transportation difficulties were significant on multivariate testing. Unstable housing is known to be a modifiable risk factor for HCV transmission [10]. Patients living with HCV who experience homelessness face unique barriers to HCV treatment, including accurate information about HCV, stigma, mistrust of the healthcare system, competing priorities (such as securing food or managing personal hygiene), and incarceration [8]. Furthermore, placement of patients with HCV experiencing homelessness into supportive housing is associated with overall reduced morbidity and mortality [11]. A lack of adequate transportation and distance to the clinic can also hinder clinic attendance [12]. To mitigate the impact of transportation difficulties, providers may deliver HCV care outside the traditional clinic [13]. Patients with a history of medication non-adherence may have faced stigma within the healthcare system and may be cautious about seeking outpatient HCV care [14]. Additionally, some patients may not seek outpatient HCV care as some providers are reluctant to start HCV treatment among patients with concerns for non-adherence [15].

Previous studies identified gaps in the HCV evaluation and treatment care among racial minorities [16]. We did not match the cohorts based on race or ethnicity to evaluate for disparities in HCV evaluation and treatment by race and ethnicity. Interestingly, we identified more Black patients and fewer White patients who attended their outpatient HCV visits on bivariate, but not multivariate, analysis. Further work among racial and ethnic minorities is needed to identify interventions to reduce health disparities in the evaluation and treatment of HCV.

We did not identify significant differences in the prevalence of substance use, mental health disorders, and alcohol use among patients who were and were not able to attend their clinic appointments, potentially due to our sample size. Substance use was the most common risk factor identified in this study. Globally, the seroprevalence of HCV among people who inject drugs (PWIDs) is estimated at 60%-80% [17]. Previous studies have noted that PWIDs can benefit from the treatment of HCV and that their treatment is cost-effective compared to delaying treatment until the development of cirrhosis [18]. Mental health disorders were also prevalent among patients who did not attend their initial HCV visit. A mental health history and appropriate referral for mental healthcare are key in engaging patients in HCV care, as treatment of underlying mental health diseases is associated with higher rates of HCV treatment initiation and treatment adherence [19]. Alcohol use is a known barrier to HCV treatment and was identified in half of the patients in this study [20].

Most patients (90%) referred to the HCV clinic attended their initial evaluation appointment. Linkage to outpatient care is a known "bottleneck" within the HCV continuum of care, as the benefits of accessible DAAs are not effective if patients are not actively engaged in HCV care [21]. Successful transitions to outpatient care depend on patient- and system-level factors and may differ across HCV centers and cities [20]. Possible interventions to aid linkage to HCV include incorporating HCV care into opioid replacement therapy (ORT) and other drug or alcohol treatment programs, primary care sites, correctional facilities, and peer programs [22,23].

Our study has several limitations. The presence of risk factors was based on electronic medical record documentation, often an imperfect reflection of SDH. Overall, risk factors may have been under-documented within the medical records. Additionally, several SDHs potentially pertinent to the HCV care cascade are not typically included in the medical record, such as income level, health literacy, employment, incarceration, parental responsibilities, and stigma [24,25]. Also, only patients scheduled for a clinic appointment were included in this study. Thus, patients not scheduled for or declined an appointment were excluded. Further, our study may not be generalizable to other settings, but it likely reflects the risk factors found in urban communities. Finally, we did not evaluate system-level barriers to HCV care that may limit the transition to outpatient HCV care [19].

## Conclusions

Patients who missed their scheduled rather than attended their HCV appointments had higher rates of housing instability, transportation difficulties, and a history of medication non-adherence. Patients referred to outpatient HCV care should be screened for social determinants of health. If patients are found to have barriers to care, they should be provided additional support as appropriate to address the social determinants of health that may limit linkage to outpatient HCV care.
